# One follicle, one egg, one embryo: a case-report of successful pregnancy obtained from a single oocyte collected

**DOI:** 10.5935/1518-0557.20200087

**Published:** 2021

**Authors:** Romualdo Sciorio, Elisavet Angelaki, Naser Al-Azemi, Abubakr Elmardi

**Affiliations:** 1 Edinburgh Assisted Conception Programme, EFREC, Royal Infirmary of Edinburgh, 51 Little France Crescent, Old Dalkeith Road, Edinburgh, Scotland, EH164SA, UK; 2 Reproductive Medicine & IVF Unit, Royale Hayat Hospital, Kuwait City, Kuwait

**Keywords:** *in vitro* fertilization (IVF), Cumulus-oocyte-complex (COC), ICSI time, Fertilization, Ongoing pregnancy

## Abstract

In this report, we present an unusual case of a couple who achieved a successful pregnancy by ICSI with a single oocyte collected. The cumulus-oocyte-complex (COC) was retrieved at 36.5 hours post trigger, and was found to be at metaphase II, when sperm injection was performed at around 39 hours post trigger. At 18 hours post injection, the single oocyte was fertilized, developed to four-cell embryo on day 2, and 8-cell grade on day 3, when it was relocated in the uterine cavity. The pregnancy yielded a positive β-hCG result. The scan performed at seven weeks, revealed the presence of one amniotic sac with a fetal heartbeat. The ongoing pregnancy has been eventless at 24 weeks of gestation.

## Introduction

Over eight million in vitro fertilization (IVF) children have been born since 1978, when Steptoe and Edwards (1978) announced the first IVF baby. Worldwide, approximately 2.5 million medically assisted reproduction (MAR) cycles are performed annually, resulting in over 500,000 deliveries. During the last four decades, IVF techniques have improved steadily, thus providing better pregnancy prospects for couples suffering from infertility. However, the *in vitro* development of human embryos is still suboptimal, and many good quality embryos fail to implant and generate a viable pregnancy (Hu *et al*., 1998).

The introduction of intracytoplasmic sperm injection (ICSI) (Palermo *et al*., 1992) has revolutionized the treatment of male infertility, in particular, for patients with low sperm counts. The ejaculate of recent fathers contains approximately 73 million spermatozoa (Esteves *et al*., 2012). According to the latest World Health Organization (WHO) manual for the examination of human semen, a sperm count of over 15 million/ml is considered normal (Cooper et al., 2010), with lower thresholds (termed oligozoospermia) associated with an increased risk of infertility. In theory, a single spermatozoon would be needed to fertilize an oocyte and generate a successful pregnancy using the ICSI procedure. Thus, in most oligozoospermic patients, sperm are retrieved successfully from their ejaculated samples collected on the day of egg retrieval.

However, the number of oocytes collected is a major determinant of IVF success, with higher yields for better outcomes (Esteves *et al*., 2019a). Thus, controlled ovarian stimulation (COS) using exogenous gonadotropins to stimulate the ovary is used to promote multifollicular development, with the aim of harvesting multiple COC, which are collected surgically. A pharmacological dose of FSH is typically used to induce the growth of multiple ovarian follicles. As follicles grow and reach the requisite size, LH exposure is provided to simulate the mid-cycle LH surge, which induces the process of oocyte maturation and subsequent ovulation (Shoham *et al*., 1995). Oocyte retrieval is precisely timed following the provision of LH exposure to retrieve mature oocytes before ovulation. LH exposure initiates the resumption of meiosis and the oocyte maturation from the “metaphase I” (MI) stage to the mature “metaphase II” (MII) stage of development. During this maturation process, the first polar body is extruded, and the oocyte reaches the MII stage, which denotes its competence to be fertilized by a spermatozoon (Voronina & Wessel, 2003). Following the LH-like exposure, the remainder of the follicle forms the corpus luteum, which produces sex steroids, particularly progesterone, to prepare the endometrium for embryo implantation (Palomba *et al*., 2015).

Given the expected low number of oocytes in patients with reduced ovarian reserve and advanced maternal age, several strategies have been applied to raise the number of follicles and oocytes collected, including the increased gonadotropin dose. The classic treatment remains the COS, using the long GnRH agonist protocol, associated with hCG trigger for oocyte maturation (Chen *et al*., 2015). Despite that, in some patients undergoing IVF, only a limited number of oocytes are retrieved after COS. In this case, the likelihood of having a fertilized oocyte and a further transferable embryo with a successful pregnancy is very low, especially in women with advanced maternal age.

## Case presentation

A married couple was referred to the reproductive medicine department at Royale Hayat Hospital, Kuwait city, for ICSI treatment in 2019. They presented with a four years’ history of male infertility and low ovarian reserve. The husband’s age was 25 years and his semen analysis showed oligo-astheno-teratozoospermia. His wife was a 39 years-old lady with regular ovulatory cycles and FSH levels of 8.79 mIU/mL, and BMI of 29. They underwent a full ICSI cycle, and we counseled the couple regarding the need for ICSI treatment given the quality of sperm in the ejaculate. The woman responded poorly to the COS. Briefly, 450 IU daily of human gonadotropins (Gonal-F, Merck, Rome, Italy) were administrated for twelve days (total gonadotrophin dose of 5.400 IU). One dominant follicle was seen on the right ovary, whereas no follicles were seen on the left ovary. Oocyte maturation was triggered by using a single injection of 15.000 IU IVF-C (Human Chorionic Gonadotropin, Ciplamed) subcutaneously. Transvaginal ultrasound-guided oocyte retrieval was carried out 36.5 hours after the trigger. Only one COC was retrieved during the oocyte pick up. On that day, the sperm concentration was 5.8 million/ml, the total motility was 25%, and the strict morphology 1%.

## Oocyte retrieval, ICSI, Embryo culture and Transfer

The cumulus-oocyte-complex (COC) was isolated from follicular fluid and then rinsed in 1.0 ml G-MOPS™ plus medium (Vitrolife, Göteborg, Sweden). Following the oocyte pick-up, the oocyte was transferred to a 1.0 ml equilibrated G-IVF™ medium (Vitrolife) at 37ºC, and 6% CO_2_, 5% O_2_ and nitrogen balance in a K-System incubator (K-System G210, CooperSurgical, Inc. USA) until the time of ICSI. We collected the sperm used for the ICSI procedure by masturbation, and processed it using the density gradient technique with the following steps: first, a gradient column was prepared by placing 0.6 ml 90% gradient media (Vitrolife, Sweden) in a centrifuge tube and an additional 0.6 ml 45% gradient media layered on top. Next, up to 1.2 ml of the semen was layered on top of the 55% layer and centrifuged at 1.600 rpm for 15 minutes. The supernatant and gradient medium just above the sperm pellet were removed and discarded. The sperm pellet was washed with 6 ml G-MOPS™ plus medium (Vitrolife, Sweden) and centrifuged at 1.400 rpm for 10 minutes. The supernatant was discarded and the pellet was resuspended in 0.1 ml G-MOPS™ plus medium. Approximately 39 hours after trigger, the oocyte was treated with hyaluronidase (80 mIU/ml) for 45-60 seconds in order to remove the surrounding cumulus cells. At that time, the oocyte was found to be at the MII stage, with clear extrusion of the first polar body.

A single spermatozoon with normal morphology and progressive motility was selected under an inverted microscope (Nikon Eclipse Ti-S, Japan), and microinjected with the use of electrohydraulic injectors (Narishige, Japan). The oocyte was kept still by using a holding micropipette at the 9 o’clock position, and the polar body was oriented at the 12 o’clock position. The injecting pipette was then gently advanced through the zona pellucida and oolemma, until the pipette was beyond the center of the oocyte; then, the sperm was gently deposited into the oocyte’s cytoplasm. The oocyte was examined for the presence of two pronuclei, and successful fertilization was confirmed at approximately 18 hours after insemination.

**Figure 1:**
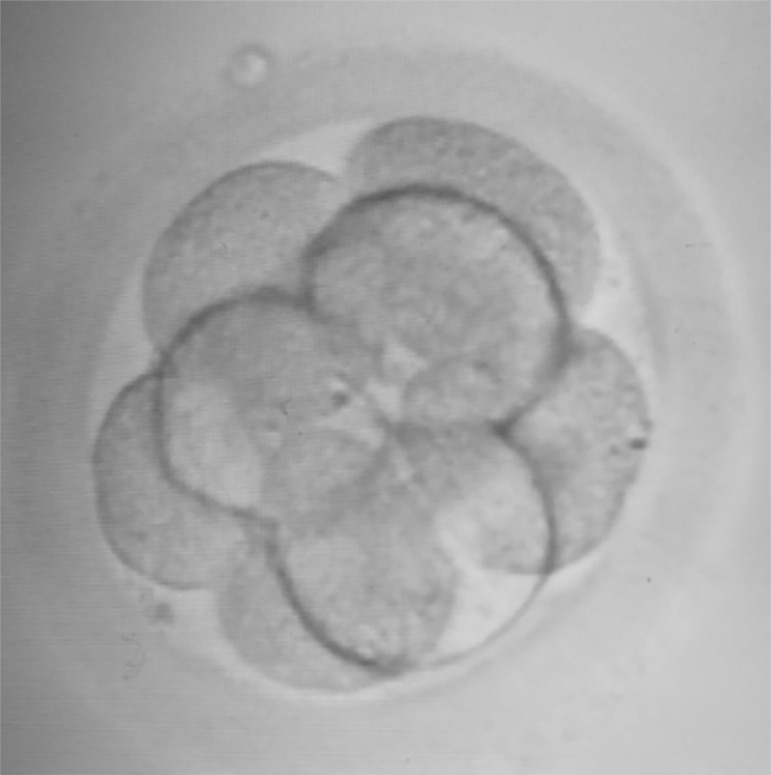
Embryo transferred on day 3

On day 2, the embryo was a four-cell grade one, reaching eight cells grade one on day 3, when it was transferred to the uterine cavity (Figure 1). The embryo culture was completed adopting a sequential pre-equilibrated medium (Vitrolife, G-series) as follows. Firstly, the fertilized oocyte was placed into a 20-microliter drop of G-1™ media, covered by light paraffin oil, sterile filtered (OVOIL™- Culture Oil, Vitrolife, Sweden). On the morning of day 3, the embryo was transferred from the G-1™ micro droplet to a 20-microliter droplet of G-2™ medium, and kept in culture until the afternoon, when the embryo transfer was performed. We hereby report a compelling case in which an ongoing pregnancy with fetal heartbeat was obtained from the only oocyte collected after COS. The embryo generated by ICSI was transferred on day 3 (Figure 1). The embryo replacement was completed under transabdominal ultrasound guidance using a soft transfer catheter (Wallace^®^ Classic, Cooper Surgical, USA). We started the luteal phase support with 90mg progesterone vaginal gel twice a day (Crinone 8%, Merck) on the evening of egg collection day and continued till the 9^th^ gestational week, adding Progylutomn tablet 2 mg, Clexane 0.4 ml and a tablet of baby Aspirin 75mg daily. At the embryo transfer, the patient started taking two injection weekly of Biosterone-Depot 250 mg and continued until nine weeks of gestation.

## Pregnancy outcome

**Figure 2:**
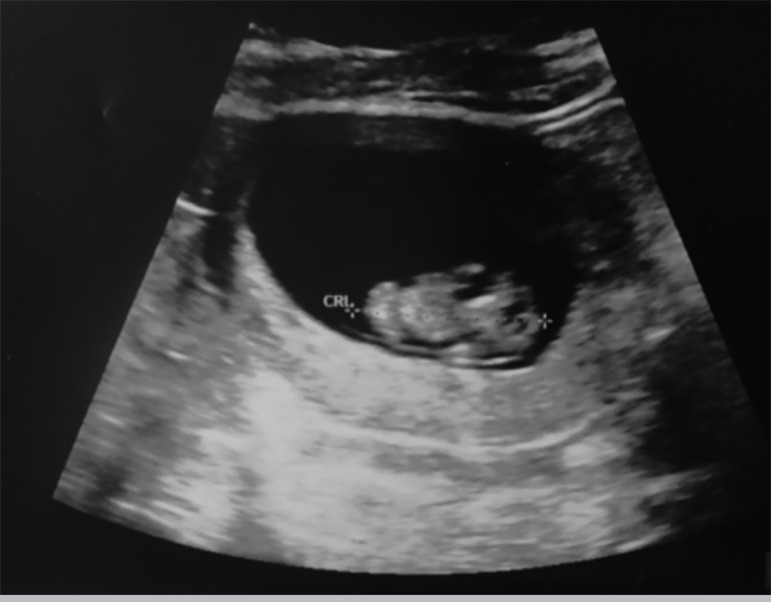
Picture shows the ultrasound scan performed at 10 weeks of gestation

We implanted the embryo and seven weeks later, we performed the first scan, which revealed the presence of one amniotic sac with fetal heartbeat (Figure 2: ultrasound scan performed at 10 weeks of gestation). Currently the gestation is at 24 weeks, and so far it has been eventless and without any complications.

## DISCUSSION

In the last few decades, the number of couples facing infertility has increased steadily, many of whom will ultimately need MAR treatments (Esteves *et al*., 2018). Global data reports over 8 million IVF babies born in the last 42 years, and in the UK, IVF babies account for about 3% of all babies born in 2016 (Adamson *et al*., 2011; Human Fertilization & Embryology Authority 2018; De Geyter *et al*., 2014). During the last 40 years, IVF techniques have improved steadily, thus providing better pregnancy prospects for couples suffering from infertility. However, despite the remarkable advances in reproductive technology, the development of human embryos *in vitro* is still suboptimal, and many good quality embryos fail to implant and generate a viable pregnancy (Hu *et al*., 1998).

IVF outcome is dependent on multiple factors, including oocyte and sperm quality, maternal age, infertility cause, lifestyle factors, as well as laboratory conditions for manipulation and embryo culture (Sciorio & Smith, 2019; Gardner, 2016). Appropriate oocyte cytoplasmic and nuclear maturation are paramount to ensure an optimal embryonic developmental competence. While nuclear maturation is usually obtained by the time of oocyte retrieval, the cytoplasmic maturation cannot be readily assessed and might be incomplete (Voronina & Wessel, 2003). Certainly, the oocyte must be at the MII stage, which denotes its competence to be fertilized by a spermatozoon. Therefore, several authors have reported that the waiting time of 2 or 4 hours between oocyte retrieval and insemination improves fertilization rate, embryo quality and pregnancy outcomes (Rienzi *et al*., 1998; Isiklar *et al*., 2004; Dozortsev *et al*., 2004).

On the other hand, excessive *in vitro* culture of human oocytes can affect their ultrastructural components, and have been linked to chromosomal and spindle abnormalities, mitochondrial damage and compromised pregnancy outcomes (Bianchi *et al*., 2015). However, a crucial aspect to notice is the oocyte number, which has a direct impact on the MAR treatment outcome. This aspect was investigated in a study published by Esteves *et al*. (2019). The authors evaluated the minimum oocyte number needed to achieve at least one euploid blastocyst for transfer. The model considered the female age, sperm source used for ICSI, and the number of mature (MII) oocytes as predictors. The final predictive model was developed using logistic regression analysis. The results found that the woman’s age the most critical predictor for the likelihood of achieving one euploid blastocyst. The estimated predicted probabilities of a mature oocyte turn into a euploid blastocyst decreased progressively with female age and was negatively affected by use of testicular sperm across age. Therefore, in this case report, we were somehow surprised to see that the only COC collected from a 39 year old lady, was found to be at MII at ICSI time, fertilized and developed into a good quality embryo, which after transfer, resulted in a viable pregnancy.

Generally, the probability of having a good quality embryo to transfer after the retrieval of a single COC is low, even lower in women with advanced maternal age (AMA; >35 years). Currently, no remedies are available to counteract the aging-related fertility decay; however, different therapeutic approaches, such as using high doses of gonadotropins to stimulate the ovary, like the one applied for the case reported here, might be offered. A recent paper published by Ubaldi *et al*., (2019) summarizes the current approaches for AMA patients undergoing MAR treatments. This case’s unique characteristic highlights the importance of MAR to overcome the most adverse situations. Health professionals providing care to infertility patients should be aware that it is possible to provide couples, like the one described here, with a real hope of biological parenthood. Equally important is to share such cases within the MAR community, which is often skeptical about the likelihood of one collected oocyte to generate a viable embryo and pregnancy with fetal heartbeat, which currently have been eventless at 24 weeks of gestation.

Finally, it is important to mention, in a case like this one, it is important that the couple have appropriate counseling. With only one follicle available and one oocyte retrieved, it is crucial to inform the couple about the probability that the oocyte may not be fertilized, leaving no embryo for transfer. Discussing these data will help set reasonable expectations and enable quick movement to a future treatment.

## CONCLUSION

We report here a compelling case in which a 39 year-old woman after COS, was able to produce one follicle. Approximately 36.5 hours after trigger, one oocyte was collected and then fertilized by ICSI. The fertilized embryo on day 3 was found to be at the eight-cell stage; it was relocated to the uterine cavity, and resulted in a successful pregnancy. Our case-report stimulates the discussion on the above aspects, and emphasize that, although live birth would have been the ultimate endpoint for such report, we do believe the ongoing pregnancy at 24 weeks to be relevant for stimulating the discussion of unusual aspects of this report.
